# Phase imaging with computational specificity (PICS) for measuring dry mass changes in sub-cellular compartments

**DOI:** 10.1038/s41467-020-20062-x

**Published:** 2020-12-07

**Authors:** Mikhail E. Kandel, Yuchen R. He, Young Jae Lee, Taylor Hsuan-Yu Chen, Kathryn Michele Sullivan, Onur Aydin, M. Taher A. Saif, Hyunjoon Kong, Nahil Sobh, Gabriel Popescu

**Affiliations:** 1 Beckman Institute, University of Illinois at Urbana-Champaign, Urbana, IL USA; 2 Department of Electrical and Computer Engineering, University of Illinois at Urbana-Champaign, Urbana, IL USA; 3 Neuroscience Program, University of Illinois at Urbana-Champaign, Urbana, IL USA; 4 Department of Bioengineering, University of Illinois at Urbana-Champaign, Urbana, IL USA; 5 Department of Mechanical Science and Engineering, University of Illinois at Urbana-Champaign, Urbana, IL USA; 6 Chemical and Biomolecular Engineering, University of Illinois at Urbana-Champaign, Urbana, IL USA; 7 Carl Woese Institute for Genomic Biology, University of Illinois at Urbana-Champaign, Urbana, IL USA

**Keywords:** Cell growth, Interference microscopy

## Abstract

Due to its specificity, fluorescence microscopy has become a quintessential imaging tool in cell biology. However, photobleaching, phototoxicity, and related artifacts continue to limit fluorescence microscopy’s utility. Recently, it has been shown that artificial intelligence (AI) can transform one form of contrast into another. We present phase imaging with computational specificity (PICS), a combination of quantitative phase imaging and AI, which provides information about unlabeled live cells with high specificity. Our imaging system allows for automatic training, while inference is built into the acquisition software and runs in real-time. Applying the computed fluorescence maps back to the quantitative phase imaging (QPI) data, we measured the growth of both nuclei and cytoplasm independently, over many days, without loss of viability. Using a QPI method that suppresses multiple scattering, we measured the dry mass content of individual cell nuclei within spheroids. In its current implementation, PICS offers a versatile quantitative technique for continuous simultaneous monitoring of individual cellular components in biological applications where long-term label-free imaging is desirable.

## Abstract (web summary)

Quantitative phase imaging suffers from a lack of specificity in label-free imaging. Here, the authors introduce Phase Imaging with Computational Specificity (PICS), a method that combines phase imaging with machine learning techniques to provide specificity in unlabeled live cells with automatic training.

## Introduction

Fluorescence microscopy has been the most common imaging tool for studying cellular biology^1^. Fluorescence signals, whether intrinsic or extrinsic, allow the investigator to study particular structures in the biospecimen with high specificity^2^. However, this important quality comes at an expensive price: chemical toxicity and phototoxicity disturb and may kill a live cell^3,4^, while photobleaching limits the extent of the investigation window^5^. Breakthroughs in genetic engineering led to the family of green fluorescent proteins, which today are broadly used in live cells with reduced toxicity^6^. In addition, current research efforts are dedicated to reducing photobleaching by various methods, including oxygen scavenging^7^ and replacing traditional fluorophores with quantum dots^8^.

Microscopy with intrinsic contrast preceded fluorescence labeling by more than two centuries^9^. Advanced forms of label-free imaging, such as phase-contrast microscopy, developed in the 1930s^10^, and differential interference contrast, in the 1950s^11^, extended the capability of imaging transparent specimens, including live cells. However, the lack of chemical specificity and inability to inform on underlying mechanisms has relegated these modalities to routine tasks, such as visual inspection of tissue cultures. Thus, fluorescence microscopy has remained a necessity for in-depth biology.

Recently, quantitative phase imaging (QPI) has advanced label-free microscopy with its ability to extract quantitative parameters (cell dry mass, cell mass transport, cell tomography, nanoscale morphology, topography, pathology markers, etc.) from unlabeled cells and tissues^12^. As a result, QPI can extract structure and dynamics information from live cells without photodamage or photobleaching^13–19^. However, in the absence of labels, QPI cannot easily identify particular structures in the cell as the label-free image lacks specificity.

In a parallel development, within the past few years, in part due to the continuous decline of computing power cost, development of frameworks for dataflow representation as well as a steep increase in data generation, deep-learning techniques have been translating from consumer to scientific applications^20–24^. For example, it has been shown that AI can map one form of contrast into another, a concept coined as image-to-image translation^25–28^, which provides a data-driven approach to estimate the fluorescence stain from unlabeled specimen. One of the first applications of this strategy was to estimate histological staining from QPI^28^ or autofluorescence^29^ signals of tissues. In those works, a separate microscope was employed to collect the stained bright-field images followed by a semi-automatic image registration step. A similar methodology was used to convert holographic microscopy images of isolated sperm cells to their virtually stained counterparts^30^. As in previous work, the stain was estimated on dead cells as a purely computational post-processing step.

Unlike the previously proposed methods focusing on fixed sperm cells or tissue, our method (PICS) can be applied to live mammalian cell cultures, in real time during multi-day imaging experiments, which are only possible through label-free imaging. In addition, due to the shared optical path between QPI and fluorescence, we generate all the PICS training data completely automatically. We applied deep learning to QPI data, generated by SLIM (spatial light interference microscopy)^31–34^ and GLIM (gradient light interference microscopy)^35,36^. These methods are white light and common path and, thus, provide high spatial and temporal sensitivity^37–44^. Because they are add-ons to existing microscopes and compatible with the fluorescence channels, these methods provide simultaneous phase and fluorescence images from the same field of view. In this way, PICS avoids the use of separate instruments such as in^28^. As a result, the training data necessary for deep learning is generated automatically, without the need for manual annotation. This augmented type of microscopy can potentially replace some commonly used tags and stains and eliminate the inconveniences associated with chemical tagging. We demonstrate this idea with various fluorescence tags and diverse cell types, at different magnifications, on different QPI systems. We show that combining QPI and computational specificity allows us to quantify the growth of subcellular components (e.g. nucleus vs cytoplasm) over many cell cycles, nondestructively. Finally, using GLIM, we imaged spheroids and demonstrated that PICS can perform single-cell nucleus identification even in such turbid structures. PICS advances the field of AI-enhanced imaging in several ways. First, PICS performs automatic registration by recording both QPI and fluorescence microscopy of the same field of view, on the same camera. The two imaging channels are integrated seamlessly by our software that controls both the QPI modules, fluorescence light path, and scanning stage. The PICS instrument can scan a large field of view, e.g., entire microscope slides, or multiwell plates, as needed. Second, PICS can achieve fluorescence channel multiplexing by automatically training on multiple fluorophores but performing inference on a single-phase image. Because PICS uses intrinsic contrast images as input, which benefit from strong signals, it provides an order of magnitude improvement in acquisition rate compared to traditional fluorescence microscopy. Third, PICS performs real-time inference, because we incorporated the AI code into the live acquisition software. The computational inference is faster than the image acquisition rate in SLIM and GLIM, which in our present implementation is 15 frames per second, thus, we add specificity without noticeable delay. To the microscope user, it would be difficult to state whether the live image originates in a fluorophore or the computer GPU. Fourth, using the specificity maps obtained by computation, we exploit the QPI channel to compute the dry mass density image associated with the particular subcellular structures. For example, using this procedure, we demonstrated a previously unachievable task: the measurement of growth curves of cell nuclei vs. cytoplasm over several days, nondestructively. Fifth, using a QPI method dedicated to imaging 3D cellular systems (GLIM), we add subcellular specificity to turbid structures such as spheroids.

## Results

### PICS method

**Fig. 1:**
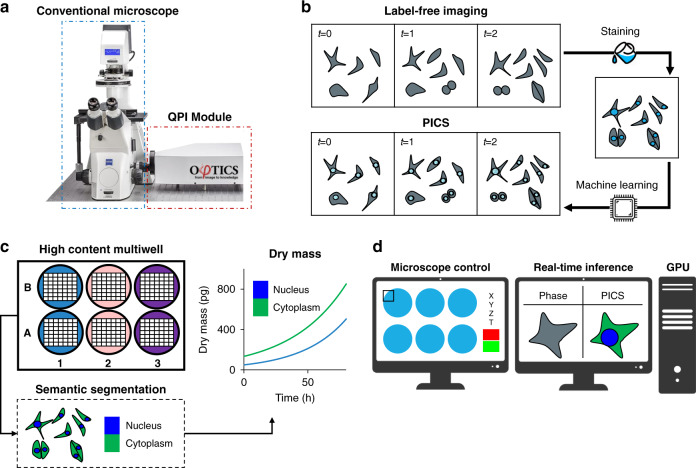
PICS method for label-free measurements of compartment-specific cellular dry mass. **a** We upgrade a conventional transmitted light microscope with a quantitative phase imaging add-on module. **b** To avoid the intrinsic toxicity of fluorescent stains, we develop a two-step protocol imaging protocol where label-free images are recorded followed by fixation and staining. From the toxic stain recorded at the end of the experiment, we train a neural network capable of digitally staining the time-lapse sequence, thus enabling time-lapse imaging of otherwise toxic stains. **c** The digital stain is used to introduce specificity to label-free imaging by providing a semantic segmentation map labeling the components of the cell. From the time-lapse sequence, we calculate organelle-specific dry mass doubling times, in this case, the rates of growth for the nucleus and cytoplasm. **d** The PICS method is integrated into a fully automated plate reading instrument capable of displaying the machine learning results in real-time.

The PICS methodology is outlined in Fig. 1. We use an inverted microscope (Axio Observer Z1, Zeiss) equipped with a QPI module (CellVista SLIM Pro and CellVista GLIM Pro, Phi Optics, Inc.). The microscope is programmed to acquire both QPI and fluorescence images of fixed, tagged cells (Fig. 1b). Once the microscope learned the new fluorophore, PICS can perform inference on the live, never labeled cells. Due to the absence of chemical toxicity and photobleaching, as well as the low power of the white-light illumination, PICS can perform dynamic imaging over arbitrary time scales, from milliseconds to weeks, without cell viability concerns. Simultaneous experiments involving multiwell plates can be performed to assay the growth and proliferation of cells of specific cellular compartments (Fig. 1c). Finally, the inference is implemented within the QPI acquisition time, such that PICS performs in real-time (Fig. 1d).

**Fig. 2:**
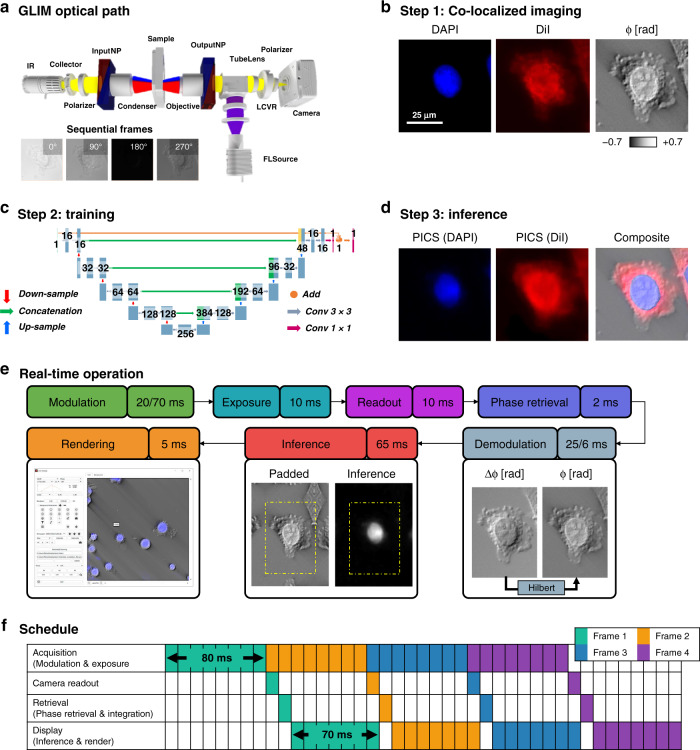
The stain location is “learned” from co-localized phase and fluorescent images. **a** Quantitative phase images are acquired with a compact Gradient Light Interference Microscopy (GLIM) module that attaches to the output port of a differential interference contrast microscope (DIC/Nomarksi). By using a liquid crystal variable retarder (LCVR) the instrument introduces controlled phase shifts between the orthogonal polarizations in DIC. GLIM images are the result of a four-frame reconstruction process to retrieve the phase associated with a differential interference contrast microscope. Insets show a zoomed portion of the field of view at 0°, 90°, 180°, 270° phase shifts. **b** The same light path is used for reflected light fluorescence imaging, providing a straightforward way to co-localize fluorescence and phase images. In this work, we focused on two popular stains used to assay the nucleus and cell body (DAPI and DiI). To recover the phase-shift associated with the object’s scattering potential, we remove the shear artifact associated with the DIC field by performing integration using a Hilbert transform to obtain *φ*, the phase shift measured along the DIC shear direction. Zoomed portion of a field of view showing a typical SW620 cell (×20/0.8). **c** Next, to learn the mapping between the label-free and stained image, we train a U-Net style deep convolutional neural network. **d** Once this model is trained we can perform real-time interference and rendering to obtain the equivalent fluorescence signal (PICS) directly from the label-free image. **e** In SLIM and GLIM, the acquisition process begins by introducing a controlled modulation which is allowed some time to stabilize (20 ms on SLIM, 70 ms on GLIM). In this work, we acquire full camera frame sizes at minimal exposure (10 ms exposure, 10 ms readout). Phase retrieval is comparably quick (2 ms, 2070 GTX Super, NVIDIA). Phase images typically require further demodulation to correct for system-specific imaging artifacts. In SLIM we perform halo removal to correct for spatial incoherence (25 ms), while GLIM images are integrated along the direction of the DIC shear (6 ms). To avoid edge artifacts, we perform GPU based inference (65 ms) on a larger, mirror padded version of the image, followed by rendering (6 ms). **f** These steps are performed in parallel to optimally overlap computation with acquisition. As the computation is typically quicker than the acquisition, during real-time operation all reconstruction steps are performed every time a new frame is received. In GLIM, the effective frame rate is limited by the modulation of the variable retarder, resulting in one phase image every 80 ms. In practice, it is possible to achieve faster performance, by using high performing graphics card and faster modulators.

PICS combines quantitative measurements of the object’s scattering potential with fluorescence microscopy. The essentials of the QPI optics and computation are shown in Fig. 2. Figure 2a illustrates the optical path of the GLIM system used for most of the QPI results in this work (for completeness, SLIM is described in Supplementary Fig. 1). The GLIM module controls the phase between the two interfering fields outputted by a DIC microscope, as described in Supplementary Note 1^35^. We acquired four intensity images corresponding to phase shifts incremented in steps of *π*/2 and combined these to obtain a quantitative phase gradient map. This gradient is integrated using a Hilbert transform method, as described in Supplementary Fig. 2 and Supplementary Note 2. The same camera records fluorescence images via epi-illumination providing a straightforward way to combine the fluorescence and phase images. Figure 2b illustrates the acquired images consisting of two fluorescence channels (cell nuclei and membrane in this case) and GLIM.

We use these co-localized image pairs to train a deep convolutional neural network to map the label-free phase images to the fluorescence data. For deep learning, we used a variant of U-Net by introducing three modifications. First, following the work by Google^45^, we added batch normalization layers before all the activation layers, which helped accelerate the training. Second, we greatly reduced the number of parameters in our network by changing the number of feature maps in each layer of the network to a quarter of what was proposed in the original paper. This change greatly reduced GPU memory usage and improved inference time, without loss of performance. Our modified U-Net model used approximately 1.9 million parameters, while the original architecture had over 30 million parameters. Based on the training results, we believe that 1.9 million parameters are sufficient to approximate the mapping from phase images to fluorescence images. Third, we utilized the advantage of residual learning^46^ with the hypothesis that it is easier for the models to approximate the mapping from phase images to the difference between phase images and fluorescence images. Thus, we implemented an addition operation between the input and the output of the last convolutional block to generate the final prediction. We noticed that this change enabled us to have much better performance under the same training conditions. The modified network architecture is shown in Fig. 2c (orange connection) and described in more detail in Supplementary Fig. 3. Figure 2d shows the result of the inference. To measure the performance of PICS under various conditions, we applied this procedure across different image resolutions, fluorophores, and cell lines, using both SLIM and GLIM (see Supplementary Fig. 4). For cellular monolayers the PICS performance is independent of confluence (Supplementary Fig. 5). Our training dataset consisted of three most in-focus images of each unique field of view, spaced 2–3 depths of field apart. This approach serves as a natural form of data augmentation. We also realized that the amount of data needed for satisfying results differ from task to task (see Supplementary Table 1).

### PICS implementation

To achieve real-time performance, we implemented our neural network using state-of-the-art libraries, optimized the network architecture, and finally modified the acquisition procedure to overlap acquisition with computation.

The PICS system uses an optimized version of the U-Net deep convolutional neural architecture to translate the quantitative phase map into a fluorescence one. To achieve real-time inference, we use TensorRT (NVIDIA) which automatically tunes the network for the specific network and GPU pairings^47^. This relatively new software package combines network operations and tunes the network operations for the active GPU. Thus, by combining kernels we avoid some kernel launch overhead and by tuning the work per element in the kernels we achieve optimal memory bandwidth. While instrumenting the layers in the C++ API was tedious, the same network without modifications runs approximately 50% faster compared to TensorFlow (conventional approach).

In the process of this work, we found that the TensorRT was unable to parse standard machine learning interchange formats such as ONNX and instead developed a script to convert the model from TensorFlow (Google) to the optimized TensorRT inference engine (NVIDIA). In short, this script transposes the weights learned by TensorFlow to match the format supported by TensorRT. In addition to performance gains, TensorRT can operate directly on GPU memory, avoiding redundant data copies and simplifying integration with existing codes.

The PICS inference framework is designed to account for differences between magnification and camera frame size. Differences in magnification are accounted for by scaling the input image to the networks’ required pixel size using NVIDIA’s Performance Primitives library. While TensorRT is fast, the network-tuning is performed online and can take a non-negligible time to initialize (30 s). To avoid tuning the network for each camera sensor size, we construct an optimized network for the largest image size and extend smaller images by mirror padding. Further, to avoid the edge artifacts typical of deep convolutional neural networks, a 32-pixel mirror pad is performed for all inference using NPP (NVIDIA, Fig. 2e).

The principal modification to the acquisition procedure was to overlap computation with hardware operation (Fig. 2e). Using the threading capabilities of C++ we divide the acquisition into four steps that are performed in parallel (Fig. 2f). One thread is assigned to coordinate the acquisition which is responsible for controlling the phase modulation hardware and initiation camera exposure. A second thread waits for the image to arrive from the camera and uploads the data to the GPU. A third thread coordinates phase-retrieval and integration. Finally, a fourth thread is responsible for running inference and rendering the image into an OpenGL buffer that can be displayed by the widget kit (Qt). Importantly this procedure prevents the graphic interface from stalling between computation events. Remarkably, we find that with this robust implementation PICS can often run faster than the underlying fluorescent signal.

### Effects of resolution on PICS performance

**Fig. 3:**
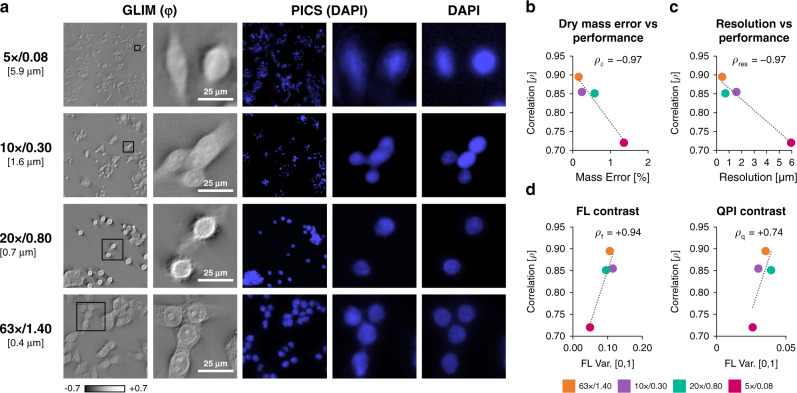
The PICS method is applicable across microscope objectives and resolutions. **a** To investigate the effect of resolution on performance we run a computational experiment where we train our network on SW cells acquired at different resolutions. To control for training sample size, we selected the most images shared between all sets. We note that the performance of the network improves with more data or training epochs. Even at low resolutions (10x, 1.6 μm resolution), we achieved adequate performance. **b** We use *ρ*, the correlation between the actual and the digital fluorescent signals over the entire set, as a quality metric. Comparing semantic segmentation maps generated form PICS and DAPI we find that even at low-resolution there is less than 2% error in estimating the dry mass. (Pearson correlation, *ρ*_ε_ = −0.97). **c** There appears to be a clear relationship (Pearson correlation, *ρ*_res_ = −0.97) between resolution (better objectives) and performance. **d** The origin of the relationship between resolution and performance is attributable to differences in contrast recorded by these objectives. We find that the *ρ*_f_ coefficient between the variance of training scaled fluorescence images and quality metric *ρ* is statistically significant while the correlation between the variance of the training scaled phase images *ρ*_q_ is weaker. Overall, these results suggest that the relationship between performance associated with resolution can be largely attributed to better overall contrast, especially for the fluorescent signal, rather than directly due to resolving capabilities.

To understand the performance of our approach we conducted a series of computational experiments where we held the training time and quantities of training pairs constant and vary the objectives used for imaging. Figure 3 shows in detail how the resolution of QPI impacts the values of the Pearson correlation coefficient that quantifies the match between the computationally predicted and actual fluorescence images. Remarkably, even for a ×5/0.08NA objective, the Pearson correlation is above 72%, which corresponds to less than 2% error when estimating the total nucleus associated dry mass of the field of view. Figure 3d presents the effect of the image contrast upon the performance. As expected, higher contrast, i.e., spatial variance, of both the fluorescence and QPI yields better performance. Note that, for a fair comparison, we kept the number of epochs constant across all resolutions and contrast, which somewhat limited the network performance (see Supplementary Table 1). A comparison between QPI, standard contrast enhancement techniques (DIC), and bright field microscopy is presented in (Supplementary Fig. 6). The data indicate that the addition of the interferometric hardware to decouple phase and amplitude information improves the performance of the AI algorithm. Furthermore, PICS provides a uniform and consistent stain. Supplementary Fig. 6 highlights a staining defect that PICS was able to correct.

### Training dataset considerations for PICS

To study the relationship between the number of training pairs vs. prediction accuracy, we conducted a second series of computational experiments where we varied the size of the dataset while keeping other training parameters constant. As shown in Supplementary Fig. 7, we found that high fidelity digital stains can be generated from as few as 20 image pairs (roughly 500 SW cells) corresponding to five minutes of training time. When the performance of our procedure is cross-validated by training on a subset of the data (Supplementary Fig. 7a), we found that certain images were dominant for training. In other words, we found that certain folds converge faster than others. Importantly, neural networks that performed well during training on small data sets (Supplementary Fig. 7a), also performed well when being validated on larger, unseen data sets (Supplementary Fig. 7b). For example, the five minutes used to train a neural network from 20 pairs is well below the time typically needed to stain the cells (Supplementary Fig. 7c).

Supplementary Fig. 8 contains a summary of the 57 networks trained for this work.

### Time-lapse PICS of adherent cells

**Fig. 4:**
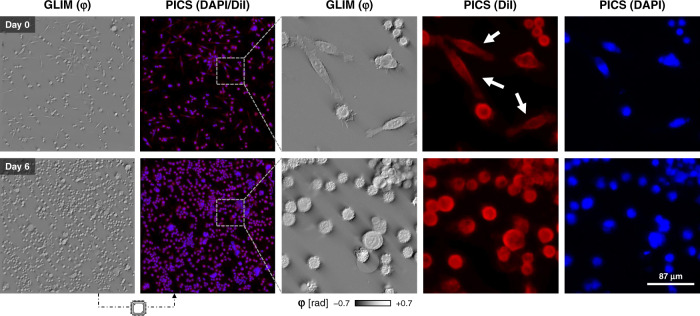
Time-lapse PICS of unstained cells. To demonstrate time-lapse imaging and high-content screening capabilities, we seeded a multiwell with three distinct concentrations of SW cells (×20/0.8). These conditions were imaged over the course of a week by acquiring mosaic tiles consisting of a 2.5 mm^2^ square area in each well using a ×20/0.8 objective. The machine learning classifier, trained at the final time point after paraformaldehyde fixation, is applied to the previously unseen sequence to yield a DiI and DAPI equivalent image. Interestingly, the neural network was able to correctly reproduce the DiI stain on more elongated fibroblast-like cells, even though few such cells are present when the training data was acquired (white arrows).

**Fig. 5:**
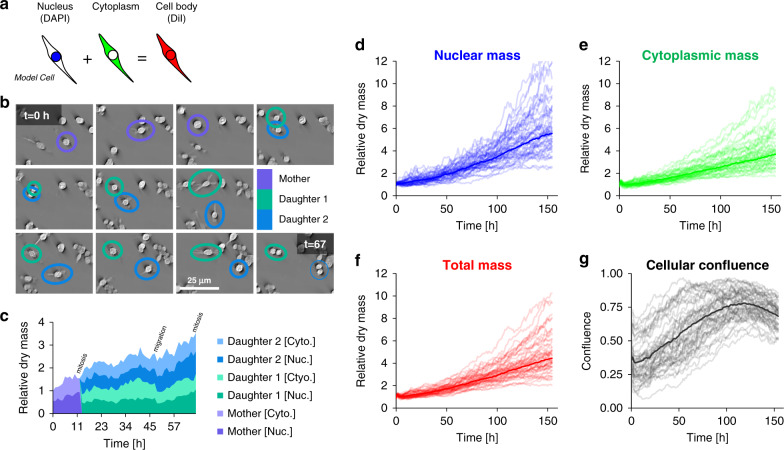
Tracking dry mass changes in cellular compartments using PICS. **a** The DiI and DAPI stains are specific to the cell body and nucleus, respectively. The difference between the two areas produces a semantic segmentation map that distinguishes between the nuclear and non-nuclear content of the cell (cytoplasm). **b** Throughout the experiment, we observe cellular growth and proliferation with cells often traveling a substantial distance between division events. **c** Using the semantic segmentation map we can track the nuclear and cytoplasmic dry mass. We find that nuclear and cytoplasmic dry mass steadily increases until mitosis, with some loss of dry mass due to cellular migration. **d**–**g** Semantic segmentation maps enable us to track the nuclear and cytoplasmic dry mass and area over 155 h. The dark curve represents the median of the growth rate across forty-nine fields of view (lighter curves). The dry mass and area are normalized by the average measured value from the first six hours. In this experiment we observe that total nuclear dry mass grows faster than total cytoplasmic mass, providing further evidence that cells can divide without growing. As the cells reach optimal confluence (*t* ≈ 114 h), we observe a decrease in the growth rate of nuclear mass, although less difference in cytoplasmic dry mass growth.

To illustrate the value of inferring multiple stains on the same cell, we acquired simultaneous PICS images of both the cell nucleus and membrane (Figs. 4, 5). Supplementary Video 1 shows the data acquisition procedure. After training, the inference model was integrated into the acquisition software for real-time operation with both SLIM and GLIM. Supplementary Videos 2 and 3 compare the real-time performance of PICS to DAPI-stained fixed cells in the same fields of view. While the final goal is to image live cells dynamically, for validation purposes, the samples were fixed, such that the chemical and estimated fluorescence signals could be quantitatively compared in real-time. Supplementary Fig. 9 describes the typical acquisition sequence and operation of the instrument. We note, that in general, fluorescence tags required an order of magnitude more exposure time than the QPI frames, implying that our plate reader achieves higher throughput while maintaining specificity. This effect is amplified when separate exposures are used for individual fluorophores.

Because of the nondestructive nature of PICS, we can apply it to monitor cells over extended periods, of many days, without a noticeable loss in cell viability. This important aspect is emphasized in Fig. 4 and Supplementary Video 4. To perform a high-content cell growth screening assay, unlabeled SW480 and SW620 cells were imaged over seven days and PICS predicted both DAPI (nucleus) and DiI (cell membrane) fluorophores. The density of the cell culture increased significantly over the seven days, a sign that cells continued their multiplication throughout the imaging. Note that, in principle, PICS can multiplex numerous stain predictions simultaneously, as training can be performed on an arbitrary number of fluorophores for the same cell type. The only price paid is computational time, as each inference channel adds, ~65 ms to the real-time inference. The computation time for one stain is completely masked by the acquisition process and multiple networks can be evaluated in parallel on separate GPUs.

### Cell growth measurements of subcellular compartments

We used PICS-DiI to generate a binary mask (Fig. 5, Supplementary Fig. 10), which, when applied to the QPI images, yields the dry mass of the entire cell. Similarly, PICS-DAPI allows us to obtain the nuclear dry mass. Thus, we can independently and dynamically monitor the dry mass content of the cytoplasm and nucleus. This capability is illustrated in Fig. 5b, c, where an individual cell is followed through mitosis. It is known that the nuclear-cytoplasmic ratio (NCR) is a controlling factor in embryogenesis^48^ and a prognosis marker in various types of cancer^49,50^. Figure 5d–f shows the specific growth curves for a large cell field of view, consisting of a mosaic of covering a 2.5 mm^2^ portion of a multiwell. Figure 5g illustrates the behavior of the confluence factor (defined as a fraction of the total area occupied by the cells) in time. Not surprisingly, as the confluence increases, the growth saturates due to contact inhibition^51^. In Supplementary Fig. 11, we repeat this imaging protocol, demonstrating that the median dry mass of the nuclei remains stable over time, while the area distinguishes between different cell lines (SW480 vs SW620). As an additional error metric, we find that cell counts taken on the training data differ on average by 6% or roughly one missed cell (typically merged neighboring cells) in every field of view. Interestingly, in this cell co-culture, while the metastatic cells (SW620) have smaller nuclei, the total dry mass is similar to that of SW480. Note that we used the same neural network for both cell lines.

### PICS of spheroids

**Fig. 6:**
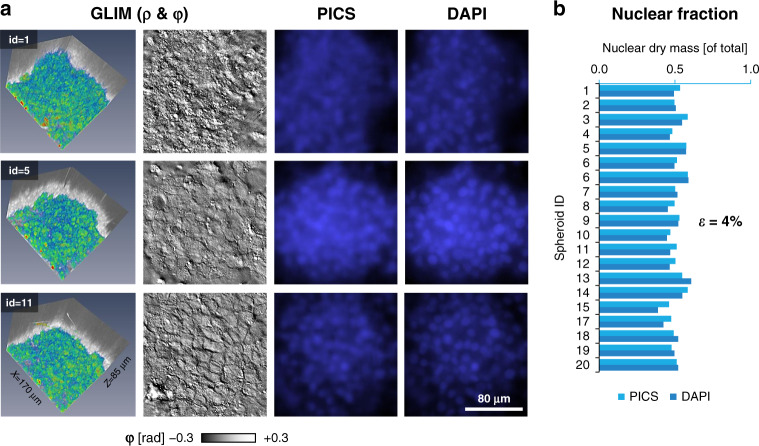
PICS for digitally staining 3D cellular systems. **a** Representative images of PICS applied to HepG2 spheroids (×63/1.4, 170 μm × 170 μm × ~85 μm). The scattering potential, GLIM (ρ) was recovered, as in the original GLIM paper, by nonlinear filtering where the absolute value of the phase map is displayed on a log scale. PICS and DAPI insets were bilaterally filtered to improve contrast. **b** To compare the performance of PICS to conventional DAPI staining we constructed a semantic segmentation map by thresholding the DAPI and PICS image and calculating the dry mass within this map. When comparing total dry mass across twenty samples, we find the average percentage change between the PICS and DAPI images to be 4%.

GLIM has been developed recently in our laboratory to extend QPI applications to thicker, strongly scattering structures, such as embryos^35^, spheroids, and acute brain slices^36^. GLIM improves image quality by suppressing artifacts due to multiple scattering and provides a quantitative method to assay cellular dry mass. To showcase this capability, we imaged 20 spheroids using GLIM equipped with a ×63/1.4NA objective. Each spheroid was imaged in depth over an 85 μm range, sampled in steps of 80 nm, with each field of view measuring 170 × 170 μm^2^. At each z-position, epi-fluorescence imaging was also performed to reveal the DAPI-stained nuclei (Fig. 6a). Following the same training procedure as before, we found that PICS can infer the nuclear map with high accuracy. Specifically, we constructed a binary mask using PICS and DAPI images and compared the fraction of mass found inside the two masks. Thus, Fig. 6b shows that the average error between inferring nuclear dry mass based on the DAPI vs. PICS mask is 4%, which is larger than for cellular monolayers, as expected (Fig. 3b).

## Discussion

PICS uses artificial intelligence for image-to-image translation to boost the capability of QPI. PICS exploits the unique capabilities of SLIM and GLIM, whereby the QPI and fluorescence images can be obtained by the same camera, without the need for complex image registration. As a result, annotation, which normally represents a bottleneck for AI, is performed automatically, with no manual intervention.

In principle, the number of fluorescent channels that PICS can predict is virtually unlimited. Our approach is to collect training data at the end of the experiment on fixed cells, effectively training for each cell type and magnification. Compared to previous efforts, we avoid using separate instruments for acquiring the fluorescence and label-free data^28^. In particular, using confocal microscopes for acquiring the fluorescence images provides better axial sectioning, at the expense of lower throughput and higher equipment cost^26^. This training is performed only once and ensures that performance is optimal. Once the training dataset is stored on the computer, the microscope user benefits from a virtual stain that can be used indefinitely. The network inference requires a mere 65 ms per frame, which is faster than the image acquisition for both SLIM and GLIM. This inference time is also approximately one order of magnitude shorter than the typical exposure in our fluorescence imaging. As a result, the specificity map is displayed in real-time and overlaid with the QPI phase map.

The main benefit of PICS over regular fluorescence is the fact that computation is, of course, nondestructive, while at the same time, QPI yields quantitative information. Furthermore, the QPI data used as input is obtained using low levels of light, which has low phototoxicity. Thus, we demonstrated time-lapse imaging of live cells over a week while maintaining cell viability intact and a high level of specificity for cellular compartments. This capability is particularly valuable when studying cell growth, which remains an insufficiently understood phenomenon^52^. In particular, we showed that by multiplexing specificity for cell nuclei and lipid bilayers, PICS can simultaneously assay nuclear, cytoplasm, and total cell growth over many cell cycles. In this way, by training on fixed cells at the end of the experiment, PICS mimics fluorescence stains (such as DAPI) that are otherwise incompatible with live-cell imaging. The approach of learning stains from fixed cells for live-cell imaging presents many opportunities. For example, there is a pressing need for developing live-cell imaging techniques capable of reproducing stains that are associated with protein expression (antibody) or membrane permeability (cell viability^53^) as these stains require fixation. While DNA probes may be difficult to infer from the optical phase images, it may be possible to track morphological changes associated with expression levels.

In PICS we boost the performance of the transmitted light image, and interestingly, found that by decoupling the amplitude and phase information, QPI images outperform their underlying modalities (phase-contrast, DIC) in AI tasks (Supplementary Fig. 6). This capability is showcased in GLIM, which provides high-contrast imaging of thick tissues by suppressing multiple scattering, enabling us to achieve subcellular specificity in optically turbid spheroids. We foresee a range of applications in this area, including viability assays in spheroids subjected to various treatments^54^.

Finally, because PICS can be implemented as a hardware add-on module to an existing phase contrast or DIC microscope with common widefield epi-fluorescence, the threshold for adaption in the field is low. The automatic training procedure allows the user to easily replace the chemical makers in their studies. The real-time inference gives instantaneous feedback about the sample, which keeps the user experience virtually unchanged while operating at an improved throughput and reduced toxicity.

As shown in Supplementary Fig. 12, PICS highlights that the quantitative phase image contains vastly more information than its fluorescence counterpart. In a broader context, PICS illustrates an alternative approach in microscopy, where the resurgence of intrinsic contrast imaging is empowered by recent advances in deep-learning methods to gain specificity.

## Methods

### Acquisition procedure

With respect to ref. ^55^, our current software was designed as a “frontend” with acquisition dialogs to generate lists of events that are then processed by a “backend”. The principal changes to the backend involved instrumenting TensorRT (NVIDIA) for real-time inference, while the frontend changes involved developing a graphic interface to facilitate plate-reader style imaging. PICS images are processed following the scheme shown in Fig. 2e. Each PICS image is the result of an acquisition sequence that collates four label-free intensity images into a phase map. The sequence begins by introducing a phase shift on the modulator (“Modulation”) followed by camera exposure and readout. In GLIM, the phase shift is introduced by a liquid crystal variable retarder (Thorlabs), which takes approximately 70 ms to fully stabilize. In SLIM a ring pattern is written on the modulator and 20 ms is allowed for the crystal to stabilize (Meadowlark, XY Series). Next, four such intensity images are collated to reconstruct the phase map (“Phase Retrieval”). In GLIM, the image is integrated (6 ms) and in SLIM we remove the phase-contrast halo artifact (25 ms). The phase map is then passed into a deep convolution neural network based on the U-Net architecture to produce a synthetic stain (65 ms). Finally, the two images are rendered as an overlay with the digital stain superimposed on the phase image (5 ms). In the “live” operating mode used for finding the sample and testing the network performance, a PICS image is produced for every intensity frame. Under typical operation, the rate-limiting factor is the speed of image acquisition rather than computation time. As a point of comparison, the two-channel fluorescence images used to train the network required approximately 1000 ms of integration time, making PICS approximately 15 times faster.

### Multiwell plate reader operation

Large samples, such as the multiwell plates (12.7 × 8.5 cm), used in this work are difficult to image due to a small but significant tilt introduced during sample placement. In this work, we compensate for sample tilt by developing a graphic user interface for plate reader applications that present each well as a 3D tomogram (Supplementary Fig. 9). Tilts are controlled by adding focus points which are used to construct a Delaunay triangulation to interpolate the plane of best focus across the mosaic tiles^55^. As the glass bottom of a multiwell is flat, we found that most tilts are linear, and good results can be achieved by focusing on the four points at the corners of the well. In addition to specifying focus points, and controlling the dimensions of the acquisition, the interface configures the microscope for multichannel acquisition with fluorescence microscopy presented alongside phase imaging. The interface presents phase imaging specific features such as modulator stabilization time and variable exposure for intensity frames.

### Training the neural networks

We chose to use the U-Net architecture^56^, which effectively captures the broad features typical of quantitative phase images. Networks were built using TensorFlow and Keras, with training performed on a variety of computers including workstations (NVIDIA GTX 1080 & GTX 2080) as well as isolated compute nodes (HAL, NCSA, 4x NVIDIA V100). Supplementary Table 1 contains a summary of the 57 networks trained in this study. No transfer learning was performed in this work. All networks were trained with the adaptive moment estimator (ADAM) against a mean squared error optimization criterion. Deep convolutional neural networks are generally sensitive to the data distribution. Namely, they are prone to wrong predictions if presented with new data that do not agree with the distribution of the training set. Thus, it is crucial to develop algorithms that can filter out out-of-distribution (OOD) data during deployment. We developed models with this filtering capability, by feeding a robust and realistic training dataset that contains not only cell cultures, but also dust particles and imaging artifacts. We believe that the inclusion of such imperfection in the training set helps the model gain some level of immunity to OOD data during deployment. For future works, we believe that adding a separate algorithm (e.g. a classification neural network) to filter OOD images (e.g., non-QPI data) can further ensure the model’s performance. Phase and fluorescence microscopy images, *I*(*x*, *y*), were normalized for machine learning as:

1 $$I_{{\mathrm{ml}}\,{\mathrm{input}}}\left( {x,y} \right) = {\mathrm{med}}\left( {0,\frac{{I\left( {x,y} \right) - \rho _{\min }}}{{\rho _{\max } - \rho _{\min }}},1} \right),$$

where *ρ*_min_ and *ρ*_max_ are the minimum and maximum pixel values across the entire training set, and med is a pixel-wise median filter designed to bring the values within the range [0,1]. Spatio-temporal broadband quantitative phase images exhibit strong sectioning and defocus effects. To address focus related issues, images were acquired as a tomographic stack. The Haar wavelet criterion from^55^ was used to select the three most in-focus images for each mosaic tile.

### Cell culture

The SW480 and SW620 pairing is a popular model for cancer progression as the cells were harvested from the tumor of the same patient before and after a metastasis event^57^. Cells obtained from ATCC were grown in Leibovitz’s L-15 media with 10% FBS and 1% pen-strep at atmospheric CO_2_. Mixed SW cells were plated at a 1:1 ratio at approximately 30% confluence.

The fluorescent lipophilic dye, DiI is used to stain the cell membrane. The application of the dye was adapted and modified from established protocol from the Thermofisher website. After the passage, mixed SW cells were allowed for two days to attach and grow in the well plate. When the cells reach the desired confluence, we prepared the staining medium by mixing 5uL of DiI labeling solution into the 1 mL of normal growth medium. We aspirated off all the previous medium on the well plate and pipetted staining medium to cover all the surface of the well plate for 20 min at 37 °C. After the incubation, we drained off the staining medium and washed the cells with warmed regular growth medium three times every 10 min. Cells were then fixed with freshly prepared 4% paraformaldehyde (PFA) for 15 min and washed with PBS two times before DAPI staining. To visualize the nucleus, DAPI was used for the experiment and DAPI solution prepared with 10uL DAPI in 10 mL PBS. The cells were incubated in DAPI solution for 10 min and washed three times with PBS before imaging.

Time-lapse microscopy was performed eight hours after platting and the slower growth rate at the start of the experiment can be attributed to the cells being in the “lag-phase” of the cell cycle^58^. The growth characteristics are consistent between experiments, suggesting that they are a constant behavior of our particular subclone.

CHO cells are commonly used for mass production of mammalian proteins^59^. CHO cells (ATCC) were cultured in Ham’s F-12 with 10% FBS and 1% pen-strep under 5% CO_2_.

HepG2 spheroids represent a kind of liver cancer that is popular for high-throughput toxicity assays. Spheroids were cultured on a glass-bottom dish as indicated in^60^, which formed spheroids at sufficiently high density. To perform an experiment typical for high-content screening, we plated cells on a poly-D-lysine coated multiwell.

As plastic affects the differential interference contrast, all cells imaged in this work were cultured on glass-bottom dishes covered with a DIC specific glass lid (P06-20-1.5-N, L001, Cellvis). While the glass lid can be avoided in SLIM imaging, using a plastic lid with GLIM will result in a total loss of interferometric contrast. All cells except the spheroids were grown on poly-D-lysine treated glass.

### Time-lapse microscopy

To illustrate the nondestructive specificity associated with PICS, we performed automated time-lapse microscopy for a week. This procedure was repeated twice (Figs. 4, 5 and again in Supplementary Fig. 11). For Figs. 4, 5, three conditions of cancer cells were plated in a 2 × 3 multiwell at five different depths. For each well, we acquire a 7 by 7 mosaic grid. This procedure is repeated for every well, with a sample taken every sixty-eight minutes. As the sample was imaged in a temperature-controlled incubator, we did not observe appreciable focus drift during the week. The resulting sequence consisted of 202,860 GLIM images, which were assembled into a mosaic by software developed in house^55^. After the experiment completed, the cells were fixed, stained with DiI and DAPI, and imaged to produce a training corpus for AI. To illustrate the value of the dry mass and area, a similar procedure was applied for Supplementary Fig. 11 except SW480 and SW620 were not mixed.

### Reporting summary

Further information on research design is available in the Nature Research Reporting Summary linked to this article.

## Supplementary information

Supplementary Information

Description of Additional Supplementary Files

Supplementary Video 1

Supplementary Video 2

Supplementary Video 3

Supplementary Video 4

Reporting Summary

## Data Availability

The data that support the findings of this study are available upon reasonable request.

## Code availability

The code and computer algorithms that support the findings of this study are available from the corresponding author upon reasonable request.
