# Supplementary material for: Phase imaging with computational specificity (PICS) for measuring dry mass changes in sub-cellular compartments
Source: Nat Commun. 2020 Dec 7;11:6256. doi: 10.1038/s41467-020-20062-x (PMC7721808; doi:10.1038/s41467-020-20062-x)
Supplement: Supplementary file 1 — Supplementary Information [file 41467_2020_20062_MOESM1_ESM.docx]

Supplementary information for

**Phase Imaging with Computational Specificity (PICS)**

**for measuring dry mass changes in sub-cellular compartments**

**Author List: Mikhail E. Kandel^1,2†^, Yuchen R. He^1,2†^, Young Jae Lee^1,3^, Taylor Hsuan-Yu Chen^1,4^, Kathryn Michele Sullivan^4^, Onur Aydin^5^, M Taher A. Saif^4,5^, Hyunjoon Kong^1,4,6,7^, Nahil Sobh^1*^, Gabriel Popescu^1,2,4*^**

1. Beckman Institute, University of Illinois at Urbana-Champaign, Urbana, IL, USA.
2. Department of Electrical and Computer Engineering, University of Illinois at Urbana-Champaign, Urbana, IL, USA.
3. Neuroscience Program, University of Illinois at Urbana-Champaign, Urbana, IL, USA.
4. Department of Bioengineering, University of Illinois at Urbana-Champaign, Urbana, IL, USA.
5. Department of Mechanical Science and Engineering, University of Illinois at Urbana-Champaign, Urbana, IL, USA.
6. Chemical and Biomolecular Engineering, University of Illinois at Urbana-Champaign, Urbana, IL, USA.
7. Carl Woese Institute for Genomic Biology, University of Illinois at Urbana-Champaign, Urbana, IL, USA.

**^†^ Equal contributions**

***Correspondence to:**

Nahil Sobh, 4039 Beckman Institute, 405 North Mathews Ave, Urbana, Illinois 61801, (217) 244-1176, sobh@illinois.edu

Gabriel Popescu, 4055 Beckman Institute, 405 North Mathews Ave, Urbana, Illinois 61801, (217) 333-4840, gpopescu@illinois.edu

**Supplementary Note 1: Gradient Light Interference Microscopy**

To show that PICS is not dependent on a particular QPI method, we used both SLIM and GLIM. GLIM is implemented as an upgrade to a conventional DIC microscope^1,2^ (Fig. 1a and Fig. 2a). To reduce photodamage and multiple scattering, we used a broadband infrared source (780 nm). The sample is illuminated by two slightly shifted fields originating from a Nomarski prism. The sample is imaged by an objective with an integrated Nomarski prism, which recombines these fields and undoes the effect of the input prism. To measure the difference in phase between the two fields, we modify the optical path by introducing a liquid crystal variable retarder (LCVR, Thorlabs) between the camera and output polarizer. The LCVR enables us to control the phase shift between the two polarizations outputted by the DIC microscope. In our instruments, we record four images corresponding to $\pi/2$ phase shifts between the two beams (Fig. 2a), which lets us recover, uniquely, the phase shift associated with the DIC microscope^1^. The resulting image resembles a derivative of the phase map associated with the object as it is based on differences in phase between neighboring points. This image is then integrated using a 1D Hilbert transform as noted in the next section. While this approach can be extended to multiple shear directions (as in ^3^), here we used only 1D integration as the phase-shifting components can be located completely outside our microscope, at the expense of certain streak artifacts in the integrated image. For GLIM, we used a 20x/0.8 NA objective giving us a sampling of roughly 0.3 microns per pixel, compared to the diffraction spot of 0.7 microns. To obtain an optimal resolution, all GLIM images were acquired with a fully open condenser (NA_c_=0.55).

**Supplementary Note 2: Phase integration using the Hilbert transform**

The phase image in GLIM is the result of interfering two laterally offset or “sheared” beams. The intensity measured at the detector resembles^2^,

[1]

where  is the gradient of the phase map. When the modulator is cycled using the liquid crystal variable retarder, we obtain the phase shift which is the derivative of the phase along the contrast direction, $x$, scaled by the shear $\delta_{x}$.

To obtain the true phase map and remove the shading effect, we perform a Hilbert transform ^4^ along the contrast direction, which performs the following Fourier filter (Supplementary Fig. 2a),

[2]

where **k** is the wavevector, and is the integrated image along the contrast direction and $\mathrm{sgn}$is the signum function. As shown in ^5^, this operation approximates an integral, which can be implemented as a Wiener filter ^6^,

[3]

where $l_{\text{reg}}$is the regularization constant. We note that our approach is a regularized version of the Wiener filtering method when $k_{x}$is large, which is also the frequency range at which the system operates with partially coherent illumination^7^.

To demonstrate the ability of the Hilbert transform to recover topographic information we imaged a 3 μm polystyrene bead embedded in immersion oil. We found that the results are in good agreement with the expected phase shift (Supplementary Fig. 2b)

[4]

The distortion orthogonal to the contrast-bearing axis (null space of the system transfer operator) bears little significance for live cell measurements, as cell shape and growth does not have a preferential direction.

**Supplementary Note 3: Spatial Light Interference Microscopy**

SLIM upgrades a phase-contrast microscope^8^ in a similar way to how GLIM upgrades DIC. In short, SLIM uses a spatial light modulator matched to the back focal plane of the objective to control the phase shift between the incident and scattered components of the optical field. Four such phase-contrast like frames are recorded to recover the phase between the two fields (Supplementary Fig. 1). Next, the total phase is obtained by estimating the phase shift of the transmitted component and compensating for the objective attenuation^9^. Finally, the “halo” associated with phase-contrast imaging is corrected by a non-linear Hilbert transform-based approach^5^.

While SLIM has higher sensitivity^10^, the GLIM illumination path performs better in strongly scattering samples and dense well plates. In strongly scattering samples, the incident light, which acts as the reference field in SLIM, vanishes exponentially^1^. In dense microplates, the transmitted light path is distorted by the meniscus or blocked by high walls^2^. GLIM and SLIM images were acquired with three different Axio Observer Z1 microscopes.

While in this work we focus on our own SLIM and GLIM methods, we expect PICS to be applicable to other modalities, especially where fluorescence can be easily overlaid with quantitative phase images^11-15^.

| 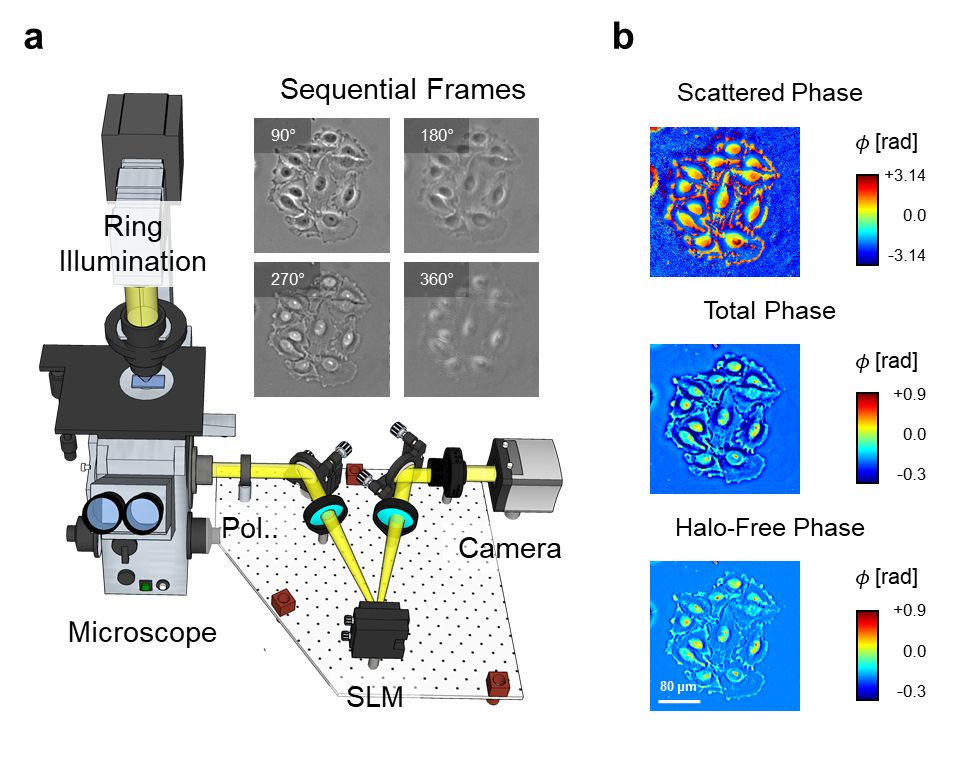 |
| --- |
| **Supplementary Figure 1: Spatial Light Interference Microscopy. a,** The ring illumination is matched to the objective’s back focal plane and the mask on the spatial light modulator (SLM), effectively resulting in a phase-contrast microscope with a variable retardance ring. Four frames are recorded, corresponding to increments of 90 degrees introduced by the SLM. **b,** SLIM image reconstruction and the halo-removed SLIM image. |

| 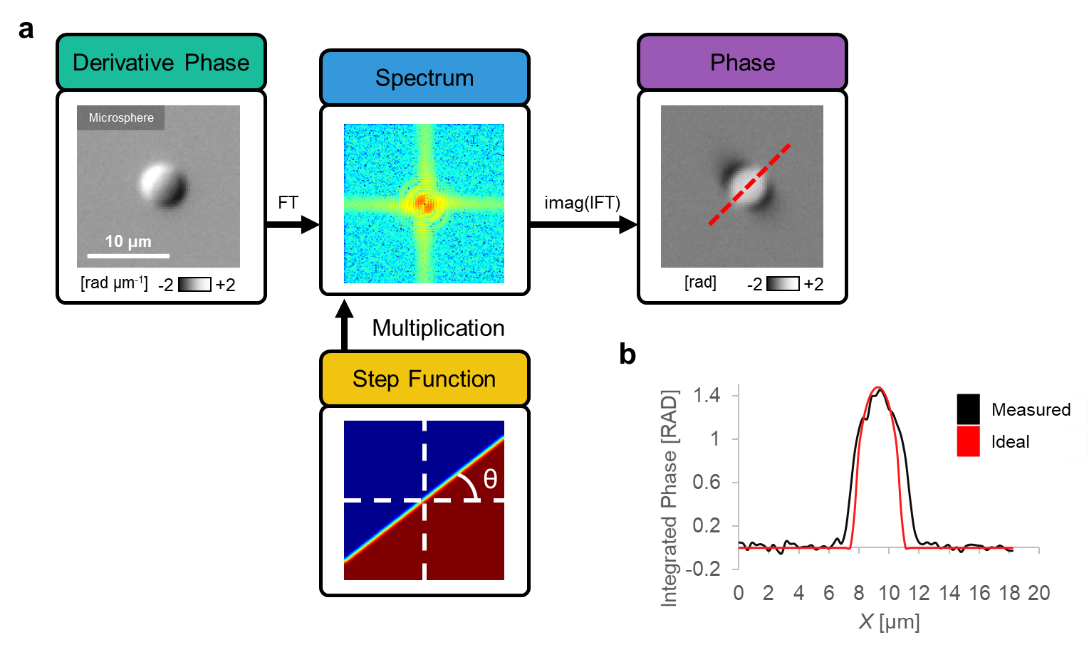 |
| --- |
| **Supplementary Figure 2: GLIM images are integrated with the Hilbert transform. a,** The Hilbert transform along the direction of the shear (θ) is performed by multiplying by a step function in the frequency domain. The imaginary portion of the inverse transform yields the integrated image. **b,** The integrated phase is in good agreement with the theoretical profile of the bead (shown on the red dashed line). |

| 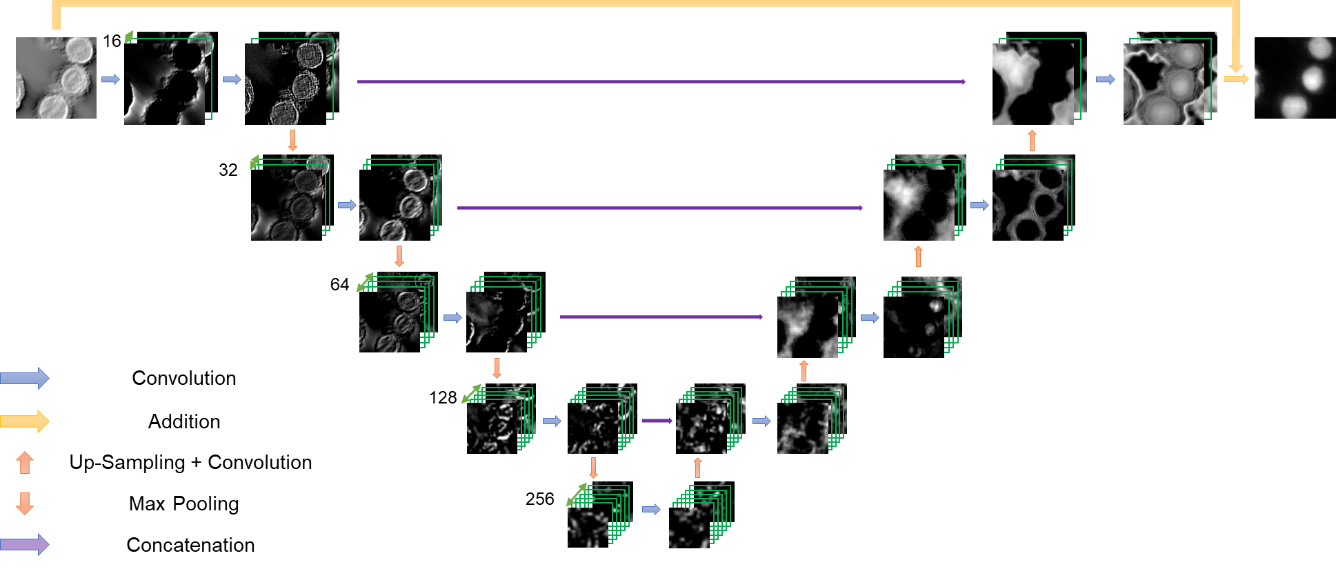 |
| --- |
| **Supplementary Figure 3: Neural Network for Phase to Fluorescence Mapping.** We modified the U-Net architecture, with batch normalization before all the activation layers and reduced the number of filters compared to the original implementation. To illustrate the evaluation of the PICS-DAPI neural network for a typical cell, we show the flow of data after applying the operations in each layer. Of particular note is the ability of the U-Net architecture to make use of both textures inside the cell (leftmost, first layers) and spatial information such as the edges around the cellular nucleolus (bottom layers). |

| 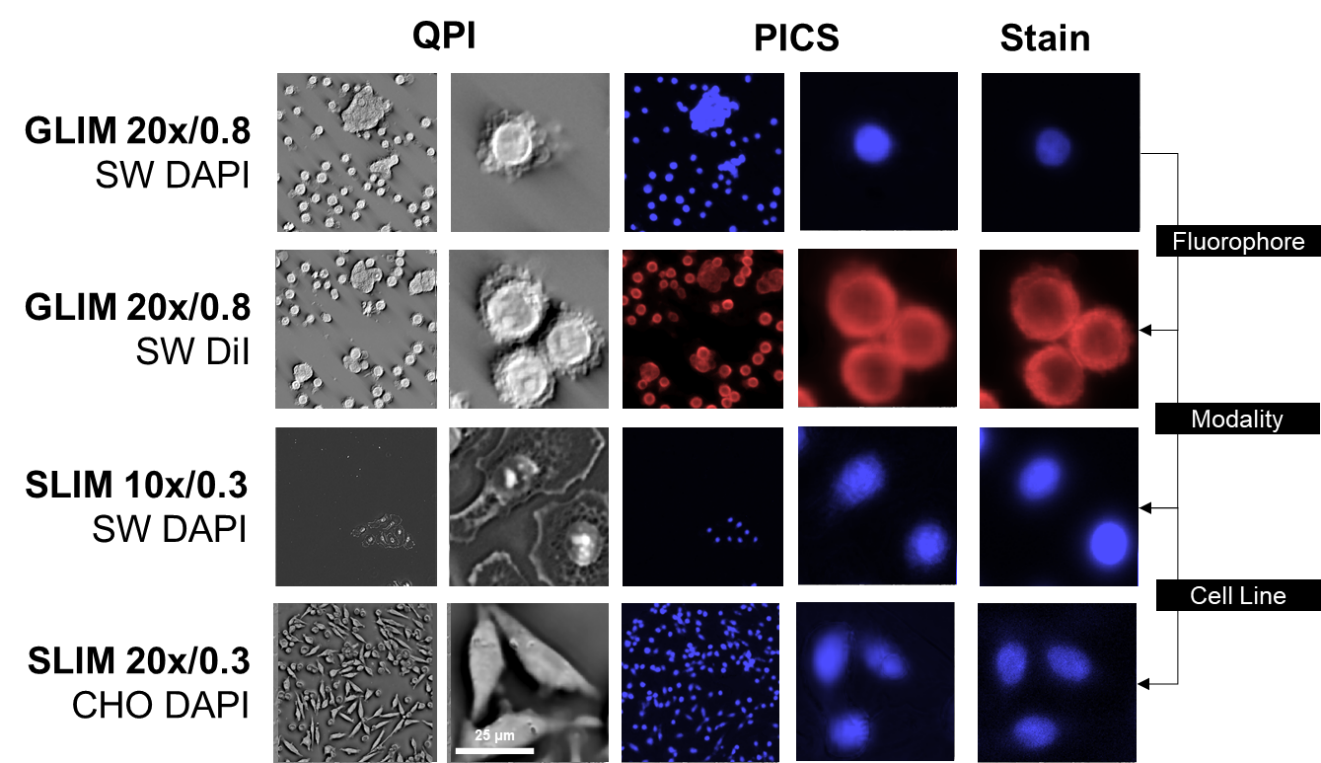 |
| --- |
| **Supplementary Figure 4: PICS method is applicable across stains, modalities, and cell-lines.** To investigate the performance of our method in various conditions, we trained separate deep convolutional neural networks on several samples and quantified their performance. As a performance metric, we compare Pearson’s correlation (ρ) between the actual fluorescence image (“Stain”) and the computationally inferred image (“PICS”). The technique is equally applicable to other QPI modalities, such as SLIM and other cell types such as CHO or a mixed culture of SW480 and SW620. |

| 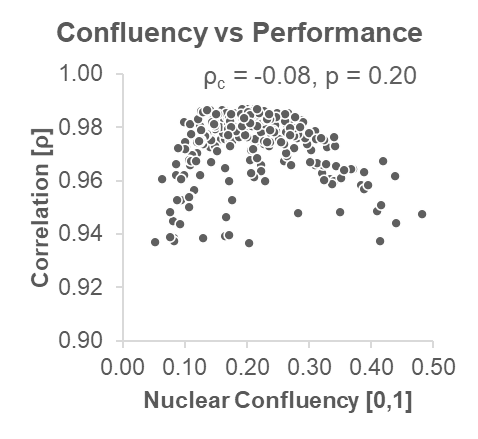 |
| --- |
| **Supplementary Figure 5: Cellular confluency is not related to PICS performance in cellular monolayers.** To investigate the role of cellular density and PICS inference performance we compared the Pearson correlation between the actual and estimated DAPI image (ρ). The comparison was performed on the training data set used in Supplementary Figure 11. Here the nuclear confluency was taken as the fraction of the mosaic tile that was occupied by the nucleus. The relationship between confluency was found to not be statistically significant (p>0.05). |

| 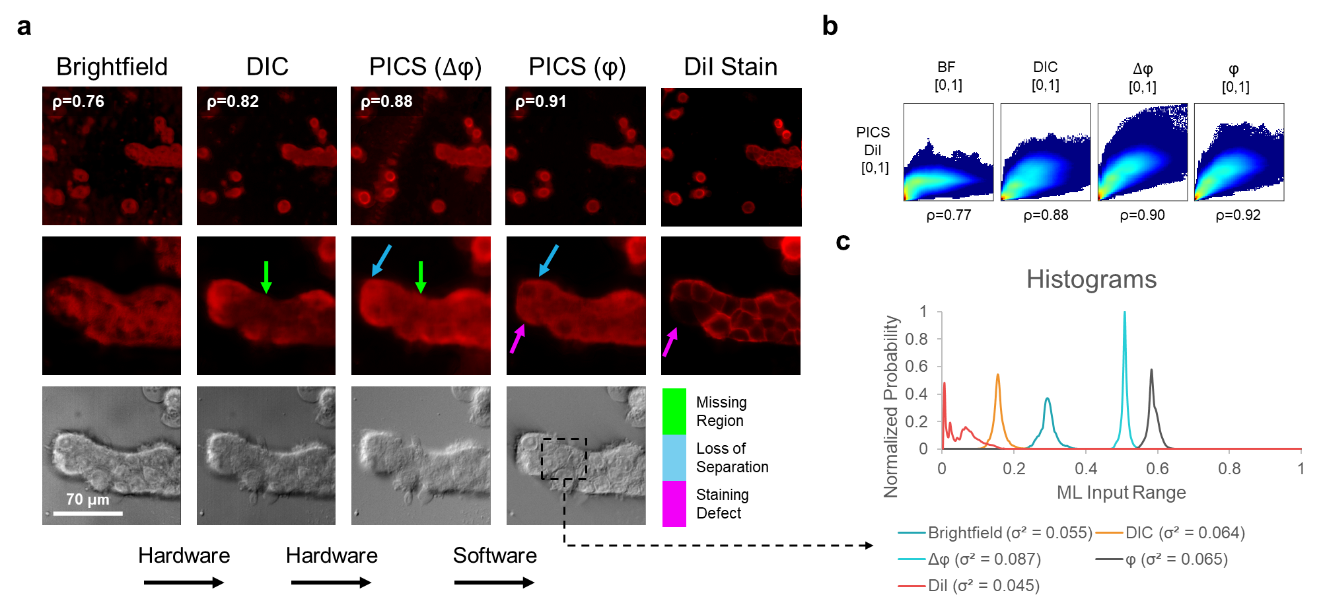 |
| --- |
| **Supplementary Figure 6: Quantitative phase information improves machine learning performance.** **a,** To compare the performance of quantitative phase imaging with conventional microscopy, we trained on different stages of the GLIM reconstruction process. Here we take the brightest GLIM frame corresponding to the least interferometric contrast as a brightfield image. DIC denotes the extinction mode frame, $\Delta\text{φ}$ is the GLIM image before integration, and $\text{φ}$ is the GLIM image after integration. As a computational experiment we train our U-Net based neural architecture on a subset of DiI images (20x/0.8), for a limited number of epochs, with the same training rate. The performance of the brightfield network is particularly poor, with an improvement when training on the DIC frame. When we introduce phase shifting ($\Delta\text{φ}$), we isolate the pure phase information resulting in a further improvement in performance (green arrows). This is especially true for at denser portions of the sample where multiple scattering contributes to unwanted amplitude information. While the ground truth image may appear sharper, the PICS neural network was able to pick up cells even when those were not fully represented due to inherent staining defects (pink arrows). **b,** To compare modalities we performed a Pearson correlation across the entire test data set, comparing the measured fluorescence to the computed fluorescent signal, showing that integrated GLIM ($\text{φ}$) has the closest match to the actual fluorescence image. **c,** To investigate the origins of these differences we plot a histogram of the image over a non-empty portion of the sample (dashed black box). When the variance inside this region is used as a contrast metric we note that comparably similar standard deviations (compare DIC at 0.064 to $\text{φ}$ at 0.065) lead to different qualitative performances. This result suggests that the difference in performance cannot be erased by simply scaling the data, rather, they are fundamental to the image formation process. |

| 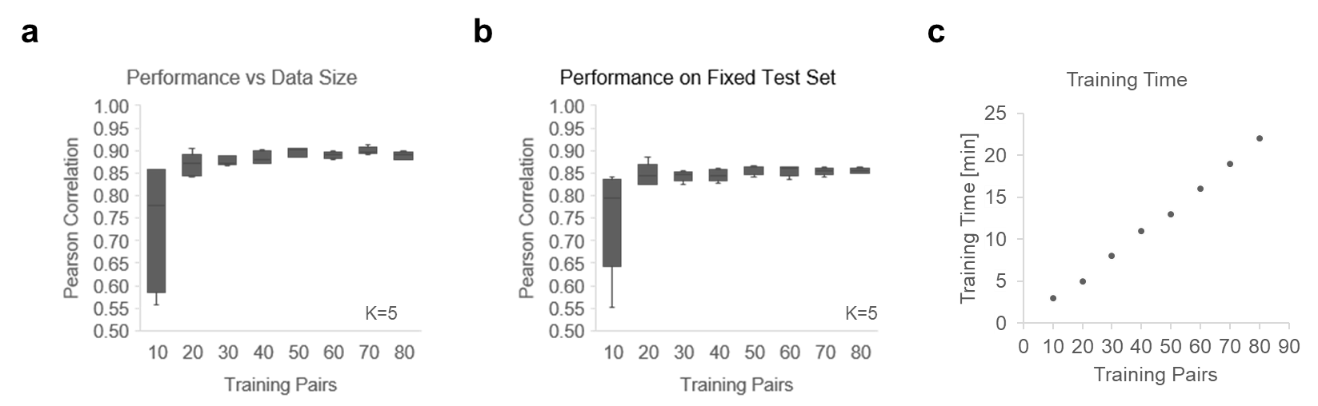 |
| --- |
| **Supplementary Figure 7: U-Net reaches asymptotic performance with a small number of training pairs.** To better understand the effects of data size on performance we conducted a numerical experiment where we trained PICS networks with an increasing number of QPI-FL training pairs. As per our convention, each “pair” consists of three focus levels, so that with 80 training pairs, we used 240 images for training, 48 for validation, and 174 for the final test group (see Supplementary Table 1). To account for differences in image selection, we perform k-fold validation (k=5), essentially training the network five times for each data set size. **a,** The performance of this network is calculated by looking at the Pearson correlation between the digital and actual fluorescent images. We note that performance becomes asymptotic, hinting that the network is fully trained after approximately 30 pairs. **b,** Each network previously trained is evaluated on an additional 58 fluorescent-phase pairs (174 phase and fluorescence images) that were not used during training. That is to say, we do no vary the test set within each k-fold. We note that this performance also becomes asymptotic after approximately 30 pairs indicating that learning the training corpus has a strong correlation to learning the transformation for unseen data. Looking at the difference in performance within k-fold validation (performance of folds within training pair 10 or 20), we note that some training pairs are substantially more performant, and this performance translates to the unseen test data. **c,** Average time to train a single fold on a single node of the HAL Cluster (NCSA). |

| 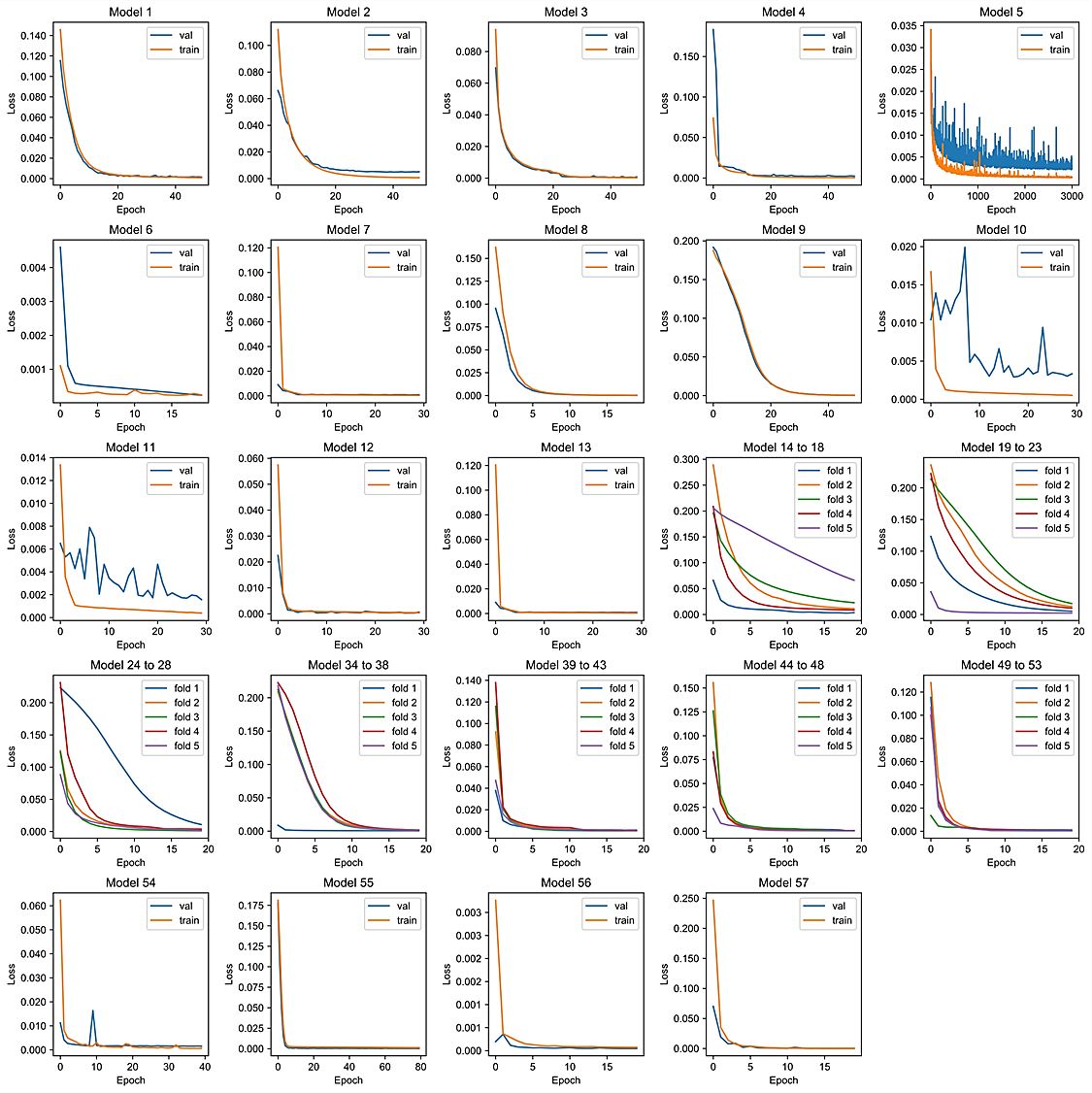 |
| --- |
| **Supplementary Figure 8: Training Plot for 57 Neural Networks.** To verify that the neural networks converged, we plot the loss on training and validation data after each epoch. A small difference between the two curves indicates that our models do not overfit. A simplified version of this plot is shown for the cross-validated networks used in Supplementary Fig. 6 showing only training loss. We note there is a substantial difference in convergence within the folds used for cross-validation, hinting that some training pairs are easier to learn than others. |

| 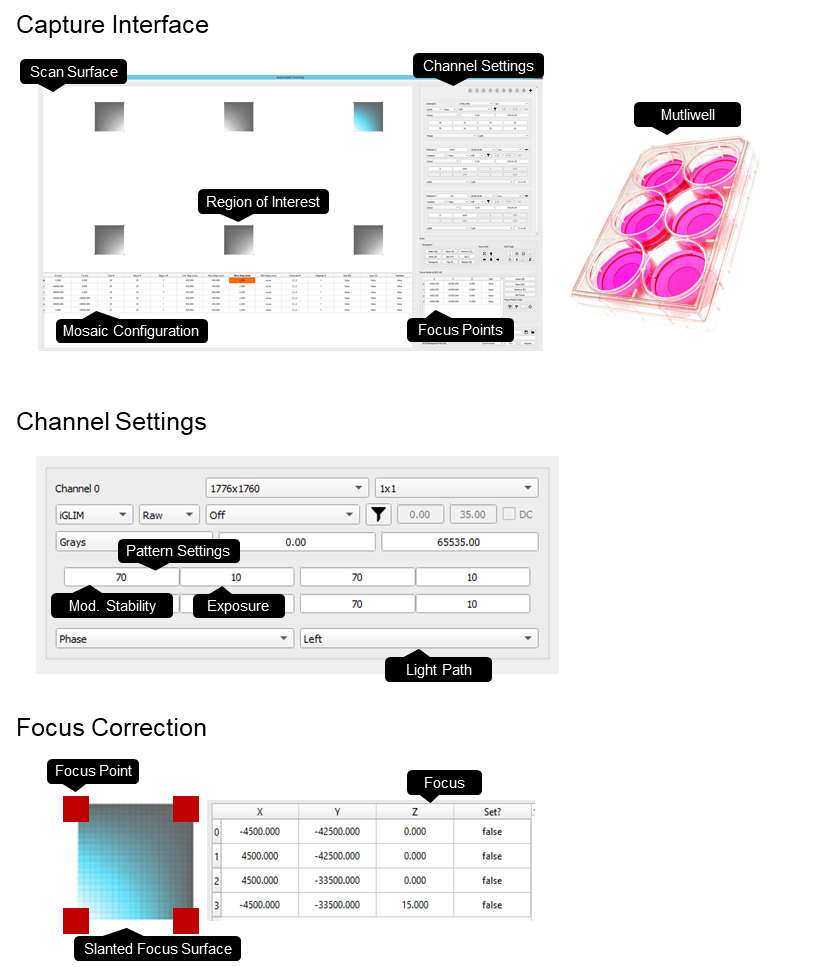 |
| --- |
| **Supplementary Figure 9: To successfully digitize multiwells we develop a graphic interface that produces a list of acquisition events that are then processed by the acquisition software.** Our capture user interface presents the multiwell as “regions of interest” (rectangles) that have associated “focus points”. The interface configures the dimensions of the volume and mosaic parameters such as the number of tiles and steps. The focus points correct for defocus in the sample (mostly due to mounting), and the scan is performed offset to the estimated tilt. In addition to configuring fluorescence acquisition, our interface contains phase imaging specific parameters such as the modulator stabilization times and exposure for each pattern used to reconstruct the phase image. |

| 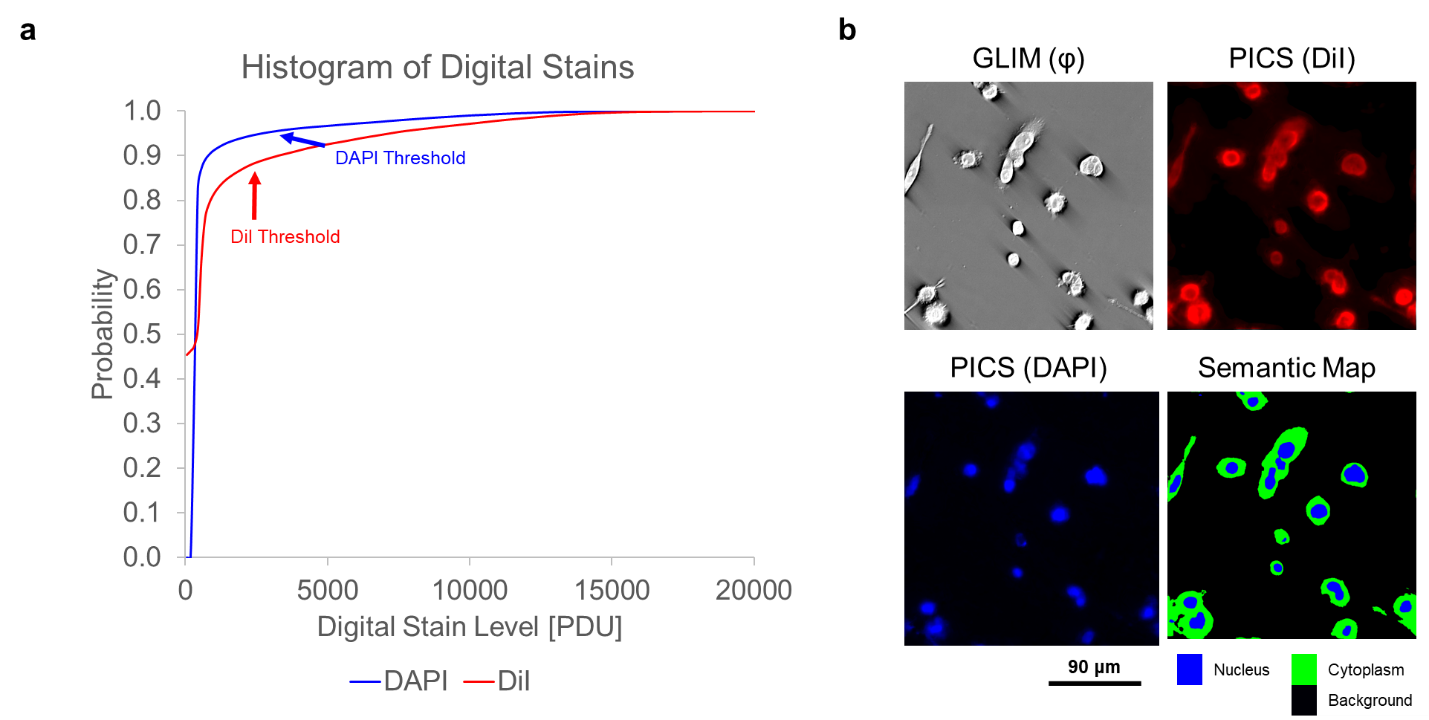 |
| --- |
| **Supplemental Figure 10: Semantic segmentation map from digital stains. a,** Digitally stained images were binarized to discriminate between stain and background by analyzing the cumulative sum of a histogram for a representative image. It was found that the change in inflection of the cumulative histogram of fluorescence intensity values served as a good threshold marker. Intuitively this change in inflection indicates when the histogram switches from tracking the background to the sample. In this work, we used the same binarization thresholds for all training pairs. **b,** Thresholded images were combined into a semantic segmentation map, by labeling all pixels with the PICS-DAPI binary mask blue, all pixels that had a PICS-DiI mask but were not blue as green (cytoplasm), and all pixels not labeled as either of the two as black (background). |

| 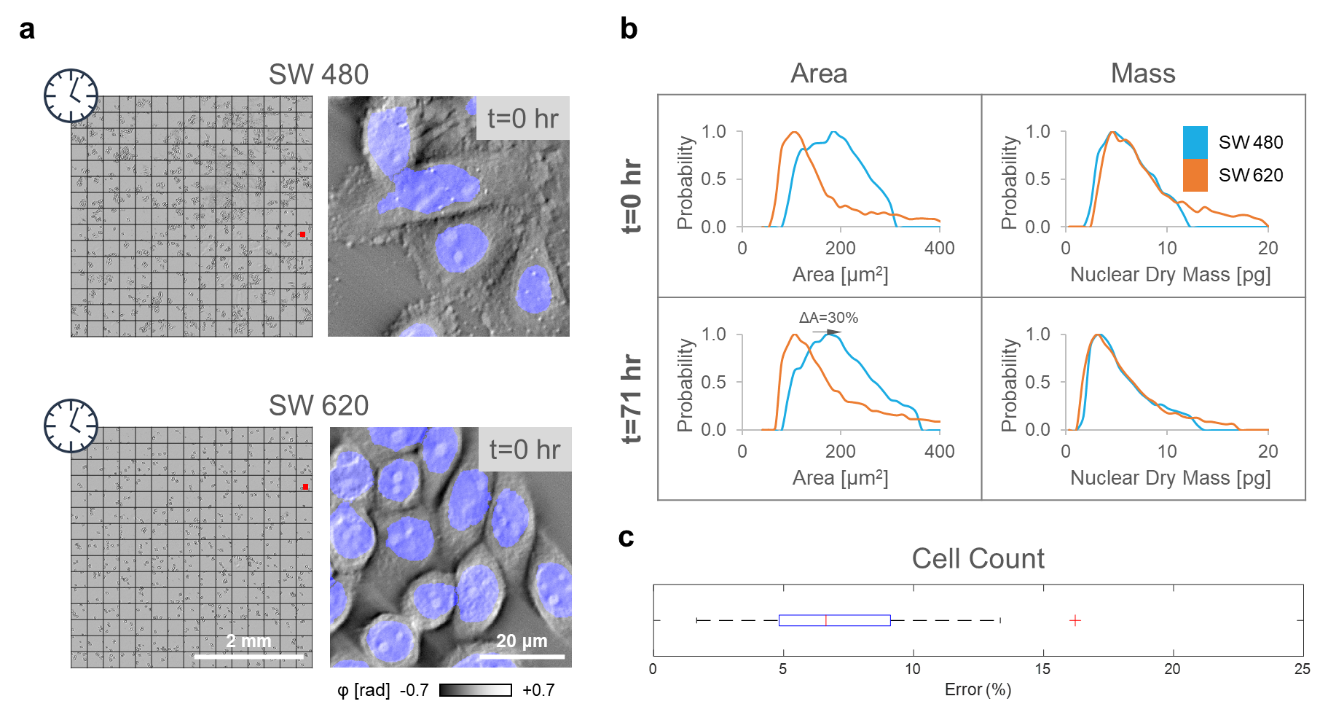 |
| --- |
| **Supplemental Figure 11: Time-lapse monitoring of nuclear dry mass and area for SW 480 and SW 620 subclones.** **a,** SW cells were images for 72 hours with a multiwell scan acquired every two hours. The semantic segmentation map from PICS was used to generate markers and ridgelines to perform instance segmentation using a watershed-based approach. **b,** instance segmentation on the cellular nucleolus shows that while SW 620 (metastatic) has somewhat smaller nuclei (ΔA=30%) total nuclear dry mass remains relatively consistent between SW 480 and SW 620. **c,** We compare the performance of PICS to the underlying fluorescence signal (DAPI) by performing a manual cell count on the pairs used to train the neural network. We find that the difference in cell counts is between PICS and DAPI is approximately 6% and can be attributed to difficulties in distinguishing touching cells. |

| 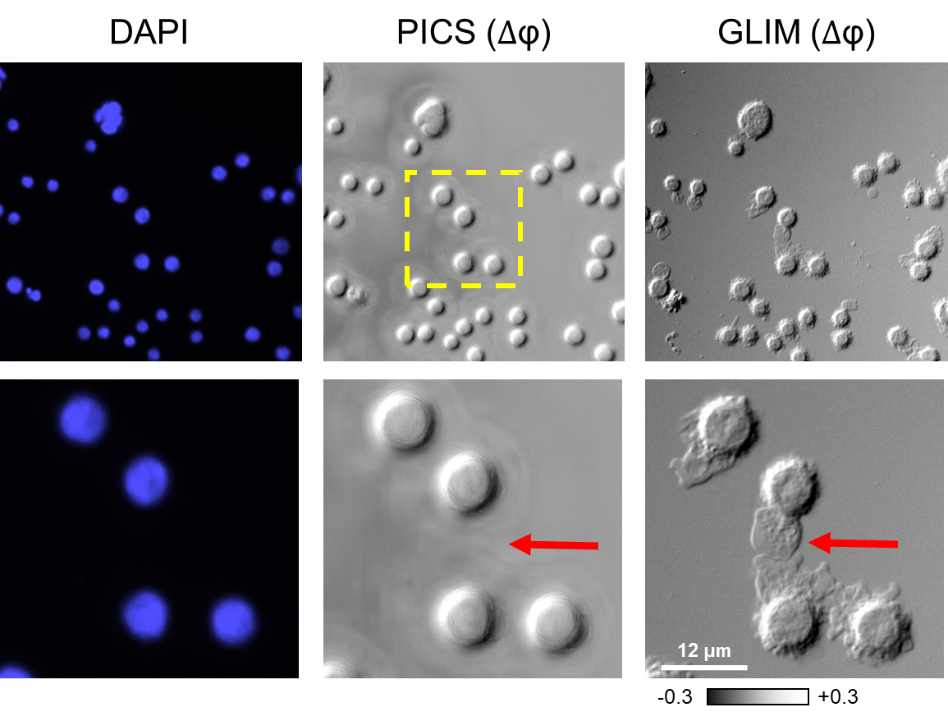 |
| --- |
| **Supplemental Figure 12: Fluorescence microscopy provides limited information compared to scattered light imaging.** While scattered light GLIM images can be used to produce fluorescence equivalents, attempting to do the reverse, going from DAPI to phase images has substantially worse performance missing structural details that would lead to a substantial underestimation of the cell’s area (red arrows). |

**Supplementary Table 1**

| **ID***** | **Appearance** | **Modality** | **Objective** | **Stain** | **Cell Line**** | **Training Pairs** | **Validation Pairs** | **Test Pairs** | **Learning Rate** | **Epochs** | **Pearson Correlation** | |
| --- | --- | --- | --- | --- | --- | --- | --- | --- | --- | --- | --- | --- |
| 1 | Fig 3 | GLIM | 05x/0.08 | DAPI | SW | 48 | 6 | 6 | 5e-5 | 50 | 0.75 | |
| 2 | Fig 3 | GLIM | 10x/0.30 | DAPI | SW | 48 | 6 | 6 | 5e-5 | 50 | 0.87 | |
| 3 | Fig 3 | GLIM | 20x/0.80 | DAPI | SW | 48 | 6 | 6 | 5e-5 | 50 | 0.89 | |
| 4 | Fig 3 | GLIM | 63x/1.40 | DAPI | SW | 48 | 6 | 6 | 5e-5 | 50 | 0.94 | |
| 5 | Fig 6 | GLIM | 63x/1.40 | DAPI | HepG2 | 16324 | 64 | 32 | 1e-4 | 3000 | 0.97 | |
| 6 | Fig S4 | GLIM | 20x/0.80 | DAPI | SW | 830 | 42 | 10 | 1e-4 | 20 | 0.93 | |
| 7 | Fig S4 | GLIM | 20x/0.80 | DiI | SW | 705 | 87 | 90 | 5e-5 | 30 | 0.94 | |
| 8 | Fig S4 | SLIM | 10x/0.30 | DAPI | SW | 210 | 30 | 30 | 1e-4 | 20 | 0.92 | |
| 9 | Fig S4 | SLIM | 20x/0.30 | DAPI | CHO | 48 | 6 | 6 | 5e-5 | 50 | 0.86 | |
| 10 | Fig S6 | BF | 20x/0.80 | DiI | SW | 705 | 87 | 90 | 5e-5 | 30 | 0.51 | |
| 11 | Fig S6 | DIC | 20x/0.80 | DiI | SW | 705 | 87 | 90 | 5e-5 | 30 | 0.87 | |
| 12 | Fig S6 | GLIM (∆φ) | 20x/0.80 | DiI | SW | 705 | 87 | 90 | 5e-5 | 30 | 0.92 | |
| 13 | Fig S6 | GLIM | 20x/0.80 | DiI | SW | 705 | 87 | 90 | 5e-5 | 30 | 0.94 | |
| 14-18 | Fig S7 | GLIM | 20x/0.80 | DAPI | SW | 30 | 6 | 174 | 1e-4 | 20 | *0.51** | |
| 19-23 | Fig S7 | GLIM | 20x/0.80 | DAPI | SW | 60 | 12 | 174 | 1e-4 | 20 | *0.65** | |
| 24-28 | Fig S7 | GLIM | 20x/0.80 | DAPI | SW | 90 | 18 | 174 | 1e-4 | 20 | *0.82** | |
| 29-33 | Fig S7 | GLIM | 20x/0.80 | DAPI | SW | 120 | 24 | 174 | 1e-4 | 20 | *0.73** | |
| 34-38 | Fig S7 | GLIM | 20x/0.80 | DAPI | SW | 150 | 30 | 174 | 1e-4 | 20 | *0.89** | |
| 39-43 | Fig S7 | GLIM | 20x/0.80 | DAPI | SW | 180 | 36 | 174 | 1e-4 | 20 | *0.86** | |
| 44-48 | Fig S7 | GLIM | 20x/0.80 | DAPI | SW | 210 | 42 | 174 | 1e-4 | 20 | *0.90** | |
| 49-53 | Fig S7 | GLIM | 20x/0.80 | DAPI | SW | 240 | 48 | 174 | 1e-4 | 20 | *0.88** | |
| 54 | Fig S5/11 | GLIM | 20x/0.80 | DAPI | SW | 390 | 45 | 45 | 1e-4 | 40 | 0.91 | |
| 55 | Fig S11 | GLIM | 20x/0.80 | DiI | SW | 3000 | 300 | 300 | 1e-5 | 80 | 0.83**** | |
| 56 | Fig S12 | GLIM | 20x/0.80 | DAPI | SW | 705 | 87 | 90 | 5e-5 | 20 | 0.78 | |
| 57 | Vid S3 | SLIM | 10x/0.30 | DAPI | SW | 210 | 30 | 30 | 1e-4 | 20 | 0.80 | |
| * Averaged performance across all k models for that k-fold cross-validation experiment  ** SW network was trained on both SW480 and SW620  *** Corresponding to IDs in supplementary Fig. 7  **** Not used for analysis | | | | | | | | | | | |

**Supplementary References**

1 Nguyen, T. H., Kandel, M. E., Rubessa, M., Wheeler, M. B. & Popescu, G. Gradient light interference microscopy for 3D imaging of unlabeled specimens. *Nat Commun* **8**, 210, doi:10.1038/s41467-017-00190-7 (2017).

2 Kandel, M. E. *et al.* Epi-illumination gradient light interference microscopy for imaging opaque structures. *Nat Commun* **10**, 4691, doi:10.1038/s41467-019-12634-3 (2019).

3 Imai, R. *et al.* Density imaging of heterochromatin in live cells using orientation-independent-DIC microscopy. *Mol Biol Cell* **28**, 3349-3359, doi:10.1091/mbc.E17-06-0359 (2017).

4 Arnison, M. R., Cogswell, C. J., Smith, N. I., Fekete, P. W. & Larkin, K. G. Using the Hilbert transform for 3D visualization of differential interference contrast microscope images. *J Microsc* **199**, 79-84, doi:10.1046/j.1365-2818.2000.00706.x (2000).

5 Kandel, M. E., Fanous, M., Best-Popescu, C. & Popescu, G. Real-time halo correction in phase contrast imaging. *Biomed. Opt. Express* **9**, 623-635, doi:10.1364/BOE.9.000623 (2018).

6 Gonzalez, R. C., Woods, R. E. & Eddins, S. L. *Digital Image Processing Using MATLAB*. (Prentice-Hall, Inc., 2003).

7 Nguyen, T. H., Edwards, C., Goddard, L. L. & Popescu, G. Quantitative phase imaging with partially coherent illumination. *Opt. Lett.* **39**, 5511-5514, doi:10.1364/OL.39.005511 (2014).

8 Wang, Z. *et al.* Spatial light interference microscopy (SLIM). *Opt. Express* **19**, 1016-1026, doi:10.1364/OE.19.001016 (2011).

9 Bhaduri, B. *et al.* Cardiomyocyte imaging using real-time spatial light interference microscopy (SLIM). *PLoS One* **8**, e56930, doi:10.1371/journal.pone.0056930 (2013).

10 Kim, T. *et al.* White-light diffraction tomography of unlabelled live cells. *Nat Photonics* **8**, 256-263, doi:10.1038/Nphoton.2013.350 (2014).

11 Park, Y., Popescu, G., Badizadegan, K., Dasari, R. R. & Feld, M. S. Diffraction phase and fluorescence microscopy. *Opt. Express* **14**, 8263-8268, doi:10.1364/oe.14.008263 (2006).

12 Chowdhury, S., Eldridge, W. J., Wax, A. & Izatt, J. A. Structured illumination multimodal 3D-resolved quantitative phase and fluorescence sub-diffraction microscopy. *Biomed. Opt. Express* **8**, 2496-2518, doi:10.1364/BOE.8.002496 (2017).

13 Yeh, L. H., Chowdhury, S. & Waller, L. Computational structured illumination for high-content fluorescence and phase microscopy. *Biomed. Opt. Express* **10**, 1978-1998, doi:10.1364/BOE.10.001978 (2019).

14 Lambert, A. Live Cell Imaging with Holotomography and Fluorescence. *Microscopy Today* **28**, 18-23, doi:10.1017/s1551929519001032 (2020).

15 Mandula, O. *et al.* Phase and fluorescence imaging with a surprisingly simple microscope based on chromatic aberration. *Opt. Express* **28**, 2079-2090, doi:10.1364/OE.28.002079 (2020).
