# Supplementary material for: Phase imaging with computational specificity (PICS) for measuring dry mass changes in sub-cellular compartments
Source: Nat Commun. 2020 Dec 7;11:6256. doi: 10.1038/s41467-020-20062-x (PMC7721808; doi:10.1038/s41467-020-20062-x)
Supplement: Supplementary file 2 — Description of Additional Supplementary Files [file 41467_2020_20062_MOESM2_ESM.docx]

**Description of Additional Supplementary Files**

**Supplementary Video 1:**

Co-localized acquisition of GLIM and DAPI data for PICS training (20x/0.8, SW cells).

**Supplementary Video 2:**

Real-time GLIM and PICS inference of DAPI (20x/0.8, SW cells).

**Supplementary Video 3:**

Real-time SLIM and PICS inference of DAPI (10x/0.3, CHO cells).

**Supplementary Video 4:**

Time-lapse GLIM and PICS imaging of unlabeled cells over seven days, with blue showing the nuclei and red the cytoplasm (20x/0.3, SW cells).
